# Hyperbolic-polaritons-enabled dark-field lens for sensitive detection

**DOI:** 10.1038/s41598-017-07576-z

**Published:** 2017-08-01

**Authors:** Lian Shen, Huaping Wang, Rujiang Li, Zhiwei Xu, Hongsheng Chen

**Affiliations:** 1 State Key Laboratory of Modern Optical Instrumentations, Zhejiang University, Hangzhou, 310027 China; 2 College of Information Science & Electronic Engineering, Zhejiang University, Hangzhou, 310027 China; 3 Institute of Marine Electronics Engineering, Zhejiang University, Hangzhou, 310058 China

## Abstract

Sensitive detection of features in a nanostructure may sometimes be puzzled in the presence of significant background noise. In this regard, background suppression and super-resolution are substantively important for detecting weakly scattering nanoscale features. Here, we present a lens design, termed hyperbolic-polaritons-enabled dark-field lens (HPEDL), which has the ability to accomplish *straightforward* sensitive detection. This HPEDL structure consists of type I and type II hyperbolic media that support high-k field waves via hyperbolic polaritons (HPs). We show that the cone-like characteristics of the HPs could be manipulated while the influence of the low-k field waves would be removed. Numerical simulations demonstrate that this proposed structure can successfully realize *straightforward* sensitive detection by modifying its thickness under the phase compensation condition. Besides, the minimum resolvable length and angular-dependent performance for sensitive detection are also demonstrated by simulations. Remarkably, these findings are very promising for propelling nanophotonics technologies and constitute a further important step towards practical applications of optical microscopy.

## Introduction

The sensitivity of microscopy to detect weakly scattering nanoscale features in the presence of significant background noise is highly limited^1^. For example, the refractive indices of the organic materials, such as Polymethyl methacrylate (PMMA) with a refractive index of 1.5 and Polycarbonate (PC) with a refractive index of 1.6, are very close to each other, so distinguishing one nanoscale organic material from another will be restricted by the diffraction limit of light and a significant background. The same problem also occurs in medical diagnosis when detecting overlapped part of the pathological field from the normal field.

On the basis of Abbe’s diffraction theory^2–4^, the imaging requires the interference of light waves scattered from the objects. Thus, in a traditional optical system, the wavelength of light sets the fundamental limit in resolving the separations between different objects. The established metamaterial-based lenses^5–21^, such as superlenses and hyperlenses, have the ability to bridge the gap between optical wavelength and nanoscale objects by transferring both propagative and evanescent waves. Nevertheless, these lenses cannot apply to suppress the background noise. To destructively interfere and eliminate the background noise, we may take advantages of the interference arrangements. Nevertheless, these schemes are understandably elaborate and intricate to figure out and therefore an optical lens that can intrinsically overcome diffraction limit and suppress a strong background would be a simpler and better solution for sensitive detection. The fundamental idea behind this kind of lens is to remove the low spatial frequencies (low-k field waves) and visualize only the fast spatial variations of the wave field (high-k field waves) created by nanoscale features. We could see that this kind of lens technique is similar in spirit to that offered by the conventional dark-field microscopy, where the significant background noise could be suppressed and the image contrast will be enhanced. Thus, such kind of lens can be classified as the dark-field lens. Recently, researchers proposed the concept of the dark-field hyperlens^22, 23^ that has the ability to enhance the image contrast. They made attempts to block low-k field waves and functions only the high-k field waves, however, there is no discussion about making image more straightforward via the cone-like pattern of the high-k field waves except for some hand-waving suggestions such as tuning dispersion relations to realize the canalization phenomenon.

In this paper, we report an alternative lens design termed hyperbolic-polaritons-enabled dark-field lens (HPEDL) that can realize *straightforward* sensitive detection. This HPEDL consisting of two types of hyperbolic media, i.e. type I (${\rm{Re}}[{\varepsilon }_{\parallel }] > 0,{\rm{Re}}[{\varepsilon }_{\perp }] < 0$) and type II (${\rm{Re}}[{\varepsilon }_{\parallel }] < 0,{\rm{Re}}[{\varepsilon }_{\perp }] > 0$), could not only block the low-k field waves but also manipulate the cone-like patterns of the hyperbolic polaritons (HPs)^24–29^ by modulating the thickness of each media. Under the condition of phase compensation, the HPEDL could be further modified and form a *straightforward* image with subwavelength details at the observation plane.

Next, we will describe the working principle of the proposed structure. Then, we will report the sensitive detection performance of the suggested HPEDL by detailed simulations, including minimum resolvable length and angular-dependent performance. Furthermore, we will extend the two-dimensional simulations to a more intuitive and realistic three-dimensional case. Finally, we will discuss the feasibility of HPEDL by using natural hyperbolic materials﻿ and﻿ recently proposed hyperbolic metasurfaces.

## Results and Discussion

### HPEDL Concept

Light emitted by or scattered from an object can be considered as a superposition of plane waves. Low-k field waves (low spatial frequencies) encode large-scale geometric features, while high-k field waves (high spatial frequencies) describe finer details. Since we focus on the subwavelength features, high-k field waves may play a key role in the later discussion. Besides, a lot of researches has shown that hyperbolic media exhibit subdiffractional and highly directional HPs^24–29^, which become a knob to turn for their proposed applications in nano-imaging and optical lithography. A moment thought will show that it is quite possible to accomplish sensitive detection by manipulating the propagation of the HPs.

**Figure 1:**
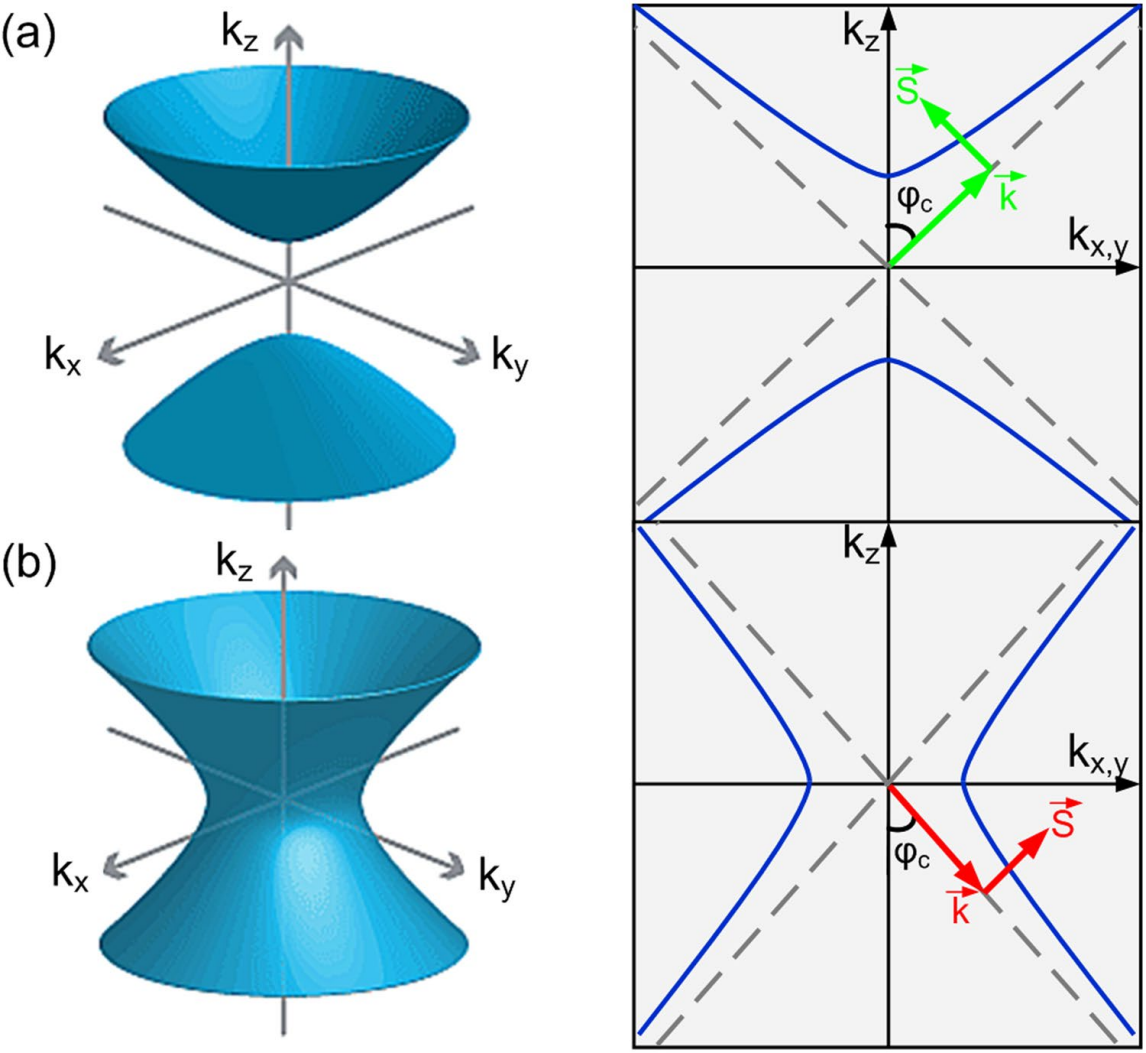
Optical behavior of hyperbolic media. Dispersion plots for (**a**) type I and (**b**) type II hyperbolic media. The arrows in the right column indicate the momentum ($\overrightarrow{k}$) and the Poynting vector ($\overrightarrow{S}$) of HPs.

We start with a general physical picture to explain the relation between the HPs and the sensitive detection. In the hyperbolic regime, the permittivity tensors (${\varepsilon }_{x}={\varepsilon }_{y}={\varepsilon }_{\parallel },{\varepsilon }_{z}={\varepsilon }_{\perp }$) have metallic characters for one of the principal components and dielectric characters for another. These particular optical properties originate from the isofrequency surface of extraordinary waves (transverse magnetic polarized), which is

1 $$\frac{{k}_{\parallel }^{2}}{{\varepsilon }_{\perp }}+\frac{{k}_{\perp }^{2}}{{\varepsilon }_{\parallel }}={k}_{0}^{2}=\frac{{\omega }^{2}}{{c}^{2}},$$

where *k*_||_ and *k*_⊥_ are the longitudinal and transverse components of the wave vector, respectively. Generally, the isofrequency surface is an open hyperboloids, of either type I (${\rm{Re}}[{\varepsilon }_{\parallel }] > 0,{\rm{Re}}[{\varepsilon }_{\perp }] < 0$, Fig. 1a), or type II (${\rm{Re}}[{\varepsilon }_{\parallel }] < 0,{\rm{Re}}[{\varepsilon }_{\perp }] > 0$, Fig. 1b). Hence, type I hyperbolic media could support the propagation of longitudinal electromagnetic waves with any wavenumber and act like dielectrics while type II hyperbolic media only support high-k propagating waves, exhibiting effective metallic properties for low-k field waves. In this regard, type II hyperbolic media can be used to remove the low spatial frequencies and act as a dark-field lens. One should notice that the hyperboloid-shaped dispersion contours result in the preferential direction of the propagation for the HPs, $\theta =\pi /2-arctan(\sqrt{{\varepsilon }_{\perp }}/i\sqrt{{\varepsilon }_{\parallel }})$ (for a more detailed analysis on HPs, please see the Supplementary Information). This typically causes waves from a point-like scatterer to propagate in a characteristic cone-like pattern.

**Figure 2:**
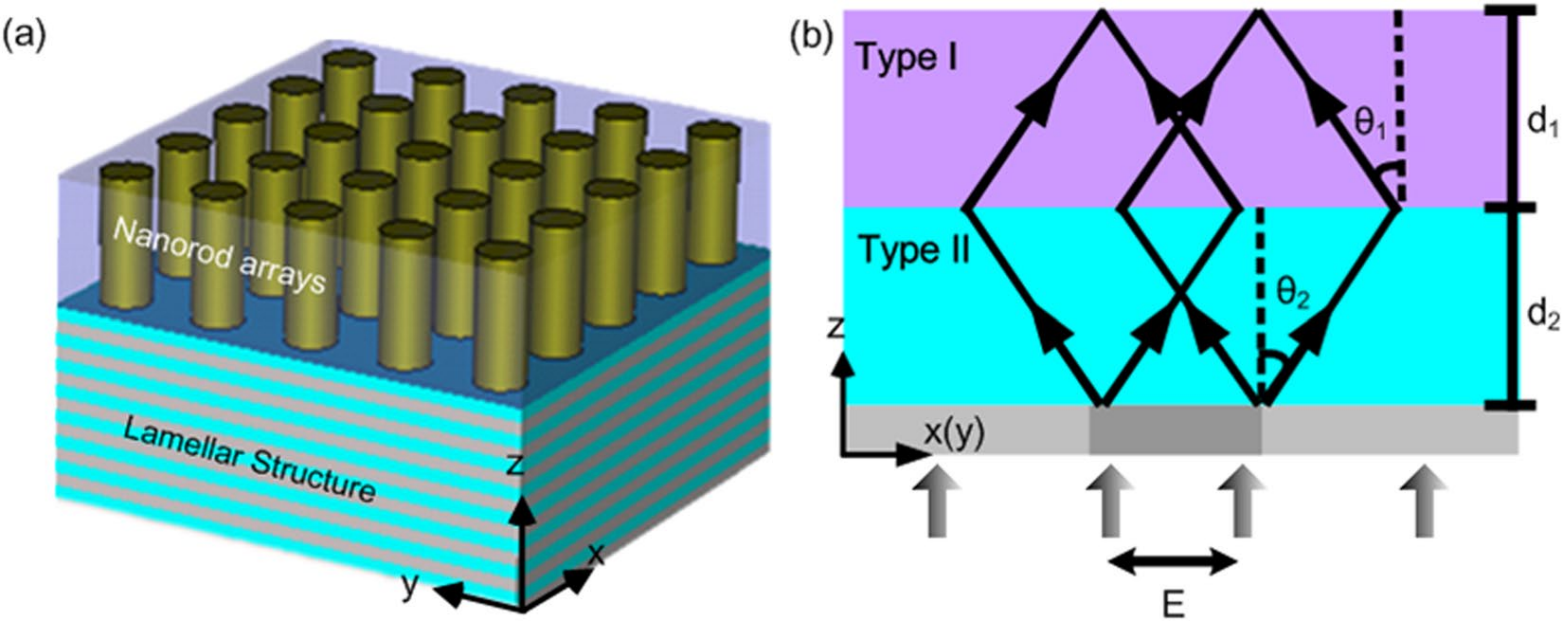
Schematic of the HPEDL and the detecting principle. (**a**) Schematic configuration of the HPEDL consisting of type I (e.g., Au nanorod arrays) and type II (e.g., Ag/SiO_2_ multilayers) hyperbolic media. (**b**) The operation principle of the HPEDL. *θ*_1_ and *θ*_2_ are the angles between the Poynting vector (dark arrows) and the optical axis for type I and type II hyperbolic media, respectively.

Consider a schematic configuration (HPEDL) that consists of type I (e.g., Au nanorod arrays) and type II (e.g., Ag/SiO_2_ multilayers) hyperbolic media as shown in Fig. 2a. From a simple ray diagram, one will find that Fig. 2b satisfies Snell’s laws of refraction at the interface as HPs inside the media make a negative angle. This will cause the cone-like HPs propagate in an inversed cone-like manner in type I hyperbolic media. Particularly, the cone-like patterns of the HPs will come to a focus when

2 $${d}_{1}\,\tan \,{\theta }_{1}={d}_{2}\,\tan \,{\theta }_{2},$$

where *θ*_1_ and *θ*_2_ are the angles between the Poynting vector and the optical axis for type I and type II hyperbolic media, respectively; *d*_1_ and *d*_2_ are the thickness of type I and type II hyperbolic media, respectively. In principle, the longitudinal component of the wave vector should be continuous at the interface. Since wave vector is orthogonal to the Poynting vector, we require that

3 $${k}_{1}\,\cos \,{\theta }_{1}={k}_{2}\,\cos \,{\theta }_{2},$$

where *k*_1_ and *k*_2_ are the wave vectors in type I and type II hyperbolic media, respectively. The phase advances in two media become ${\varphi }_{1}={k}_{1z}{d}_{1}$ and ${\varphi }_{2}={k}_{2z}{d}_{2}$, where ${k}_{1z}=-{k}_{1}\,\sin \,{\theta }_{1}$, ${k}_{2z}={k}_{2}\,\sin \,{\theta }_{2}$. The underlying secret of this structure is that the phase advances in these two media happen to be equal and opposite. In this regard, we have

4 $${\varphi }_{1}+{\varphi }_{2}=0,$$

which is also called the phase compensation condition, indicating that the HPEDL holds promise for one-to-one *straightforward* sensitive detection (for a more detailed analysis of this relation, please see the Supplementary Information). One should notice that phase compensation condition impacts more strongly on the detecting performance than impedance matching condition. Therefore, in order to achieve one-to-one *straightforward* sensitive detection, we could determine the thickness of each slab with relation (2). Unlike an earlier work^18^ that focused on the nonmagnetic process for subwavelength imaging, here we combine this method with the promising concept of HPs and bring it to the realm of dark-field.

### Visualization of Subwavelength Features

**Figure 3:**
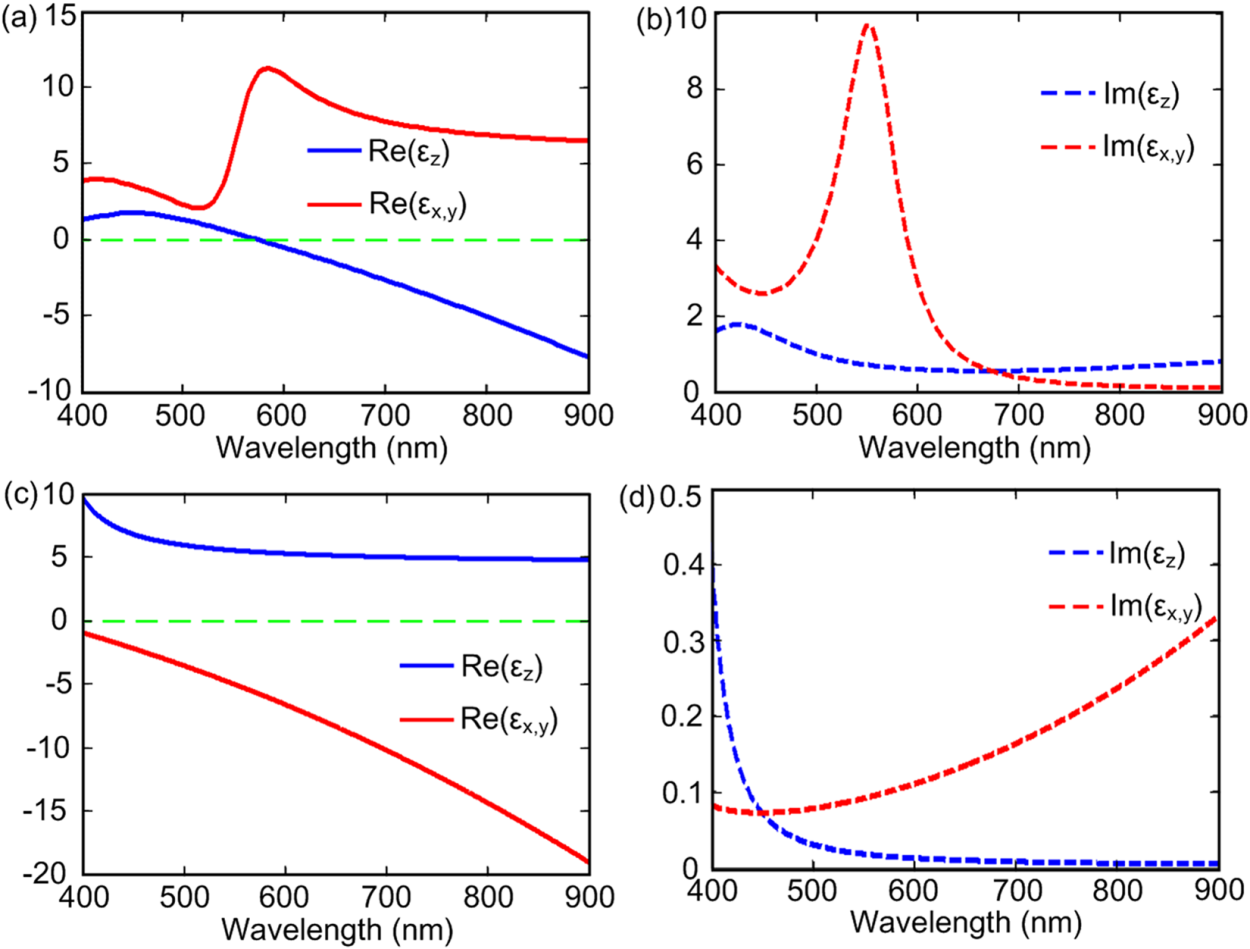
Dielectric functions of type I and type II hyperbolic metamaterials. (**a**) Real and (**b**) imaginary parts of the dielectric functions of gold nanorod arrays embedded into the Al_2_O_3_ host (*p* = 0.3). (**c**) Real and (**d**) imaginary parts of the dielectric functions of a silver-silica lamellar structure (*f*_*m*_ = 0.5). In the overlapping spectral range 575–900 nm, both type I and type II HMMs are realizable. Green dashed lines separate the positive and negative values of the real parts of the dielectric functions.

The HPEDL requires type I and type II hyperbolic media to be realizable at one wavelength. They have already been explored through artificial hyperbolic metamaterials (HMMs), such as binary metal/dielectric layers^30^ or metal nanowires in a dielectric host matrix^31, 32^. Here, we present one example of HPEDL: a type I HMM can be realized with Au nanowire arrays in Al_2_O_3_ host while a type II HMM can be achieved by alternating layers of Ag/SiO_2_ thin films. The Au nanowire arrays with average radius *r* can be fabricated by electrodeposition within nanoporous aluminum oxide membranes. Assuming that the center-to-center separation between each nanowire is *a*, in the effective medium regime where $r < a\ll {\lambda }_{0}$ with *λ*_0_ being the free-space wavelength, the optical properties of this metamaterial can be well described by averaged geometric parameters (concentration *p* = *πr* ^2^/*a* ^2^ = 0.3). Besides, the multilayer structure consisting of 8 alternating layers of Ag and SiO_2_ with each being 10 nm thick, can be subsequently deposited on a glass substrate using electron-beam evaporation, to form an overall thickness of 160 nm. By considering the optical properties of the constituent materials and their filling fractions *f*_*m*_, (filling fractions *f*_*m*_ = *a*_*m*_/(*a*_*m *_+ *a*_*d*_) = 0.5), where *a*_*m*_ and *a*_*d*_ are the thickness of individual layer of Ag and SiO_2_, respectively. The effective material permittivities of this metamaterial can be obtained by effective-medium theory (EMT). (for a more detailed analysis of the structures, please see the Supplementary Information). Figure 3 shows the dielectric functions of type I (Fig. 3a) and type II (Fig. 3b) HMMs in the spectral range 400–900 nm. Thus, type I and type II HMMs can be simultaneous realizable in the overlapping spectral range 575–900 nm. Once the permittivity tensors are determined, the propagation angles of HPs can be deduced and further the thickness of each media can be identified based on relation (2). Such design process can also be applied to different wavelengths within the overlapping spectral range 575–900 nm.

**Figure 4:**
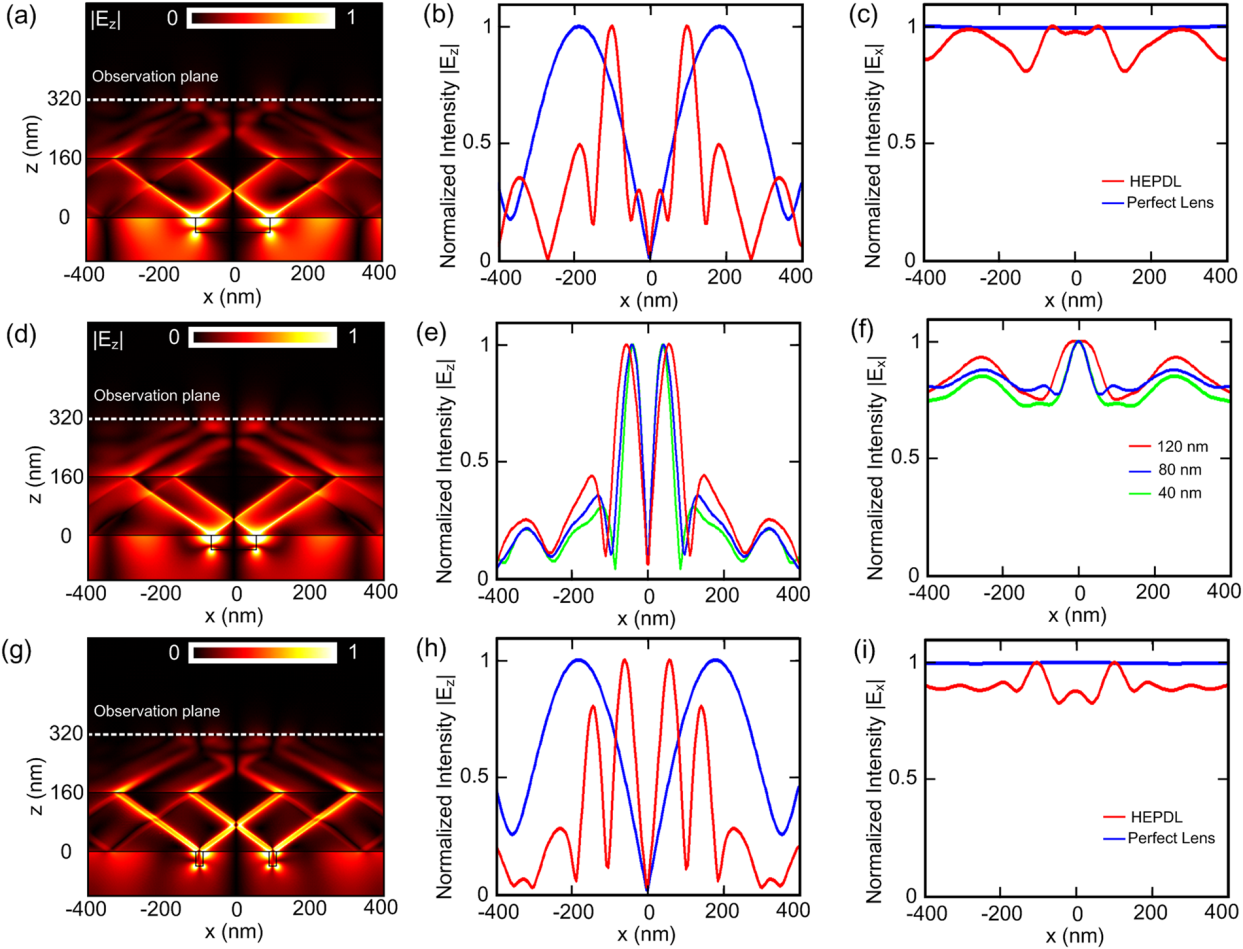
Detecting performance of the HPEDL in two-dimensional simulations. The simulations are performed at a nominal wavelength of λ_0_ = 680 nm and the thickness of the HPEDL is 320 nm (*d*_1_ = 160 *nm*, *d*_2_ = 160 *nm*). (**a,b,c**) A PMMA strip with 200 nm width that embedded in the PC substrate. (**a**) Simulated electric-field distribution |*E*_*z*_| for detecting the PMMA strip with 200 nm width. (**b**,**c**) The line profiles of (**b**) |*E*_*z*_| and (**c**) |*E*_*x*_| at the observation plane. The case of the perfect lens is shown for comparison. (**d**–**f**) PMMA strips with different widths: 120 nm, 80 nm and 40 nm. (**d**) Simulated electric-field distribution |*E*_*z*_| for detecting PMMA strip with 120 nm width. (**e,f**) The line profiles of (**e**) |*E*_*z*_| and (**f**) |*E*_*x*_| at the observation plane. (**g**,**h**,**i**) Two PMMA strips with 20 nm width and a center-to-center separation of 200 nm. (**g**) Simulated electric-field distribution |*E*_*z*_| for detecting two PMMA strips with 20 nm width 20 nm and a center-to-center separation of 200 nm. (**h**,**i**) The line profiles of (**h**) |*E*_*z*_| and (**i**) |*E*_*x*_| at the observation plane. The case of the perfect lens is shown for comparison.

To demonstrate the detecting ability of the proposed HPEDL, we utilize the frequency domain solver in COMSOL Multiphysics to perform numerical simulations for HMMs, which contains two types of media with the same thickness as *d*_1_ = *d*_2_ = 160 nm. Here, we use the HPEDL to detect a PMMA strip with 200 nm width that embedded in the PC substrate at wavelength λ_0 _= 680 nm. The simulated electric-field distribution |*E*_*z*_| is shown in Fig. 4a, from which, we could see that the electric fields *E*_*z*_ induced by the interfaces between the PMMA strip and PC substrate are effectively transferred by the HPEDL, and thus interfaces are easily distinguished. Figure 4b illustrates the line profile of |*E*_*z*_| at the observation plane. From this figure, we could find that two well-separated peaks are clearly resolved, demonstrating improved subwavelength resolution and image contrast compared with the case of ﻿the﻿ perfect lens. This is because the perfect lens supports both low- and high-k field waves, which affect the imaging performance and lead to a lower image contrast. As known from scattering theory^33, 34^, the scattered electric fields along *z* direction at interfaces between the PMMA strip and PC substrate suffer obvious variations while the scattered electric fields along *x* direction change mildly. Therefore, we could expect two weakly visible peaks for the simulated electric-field distribution |*E*_*x*_| as shown in Fig. 4c.

Since the transmission coefficient is close to unity for HPs, ideal HPEDLs could have nearly unlimited resolution. In practical, material losses associated with the thickness of the structure will relax the restriction of HPs, which may limit the performance of sensitive detection. The next question is what happens to the detecting performance when decreasing the width of the PMMA strip. The corresponding results are shown in Fig. 4d–f. Figure 4d illustrates the simulated electric-field distribution |*E*_*z*_| when the width of the PMMA strip is chosen to be 120 nm. The two peaks in Fig. 4e can still determine the exact position of each interface. However, the two weakly visible peaks for the simulated electric-field distribution |*E*_*x*_| disappear as shown in Fig. 4f. By further decreasing the width of the PMMA strip, we could find that the peaks in Fig. 4e and f will shift to a fixed position with a length scale of 80 nm. In a word, |*E*_*z*_| can be measured to identify the interfaces of the PMMA strips with width that is larger than 80 nm while |*E*_*x*_| can be utilized to determine the positions of the PMMA strips with width that is smaller than 80 nm. Figure 4g shows the simulated electric-field distribution |*E*_*z*_| when two PMMA strips with 20 nm width are embedded in the PC substrate. The separation of the centers of the two strips is 200 nm. From the line profile of the electric-field distribution |*E*_*x*_| at the observation plane, as shown in Fig. 4i, two-well separated peaks can be well resolved and utilized to describe the position of each PMMA strip as discussed. It should be noted that there will be a minimum separation between these two PMMA strips which is about 80 nm.

**Figure 5:**
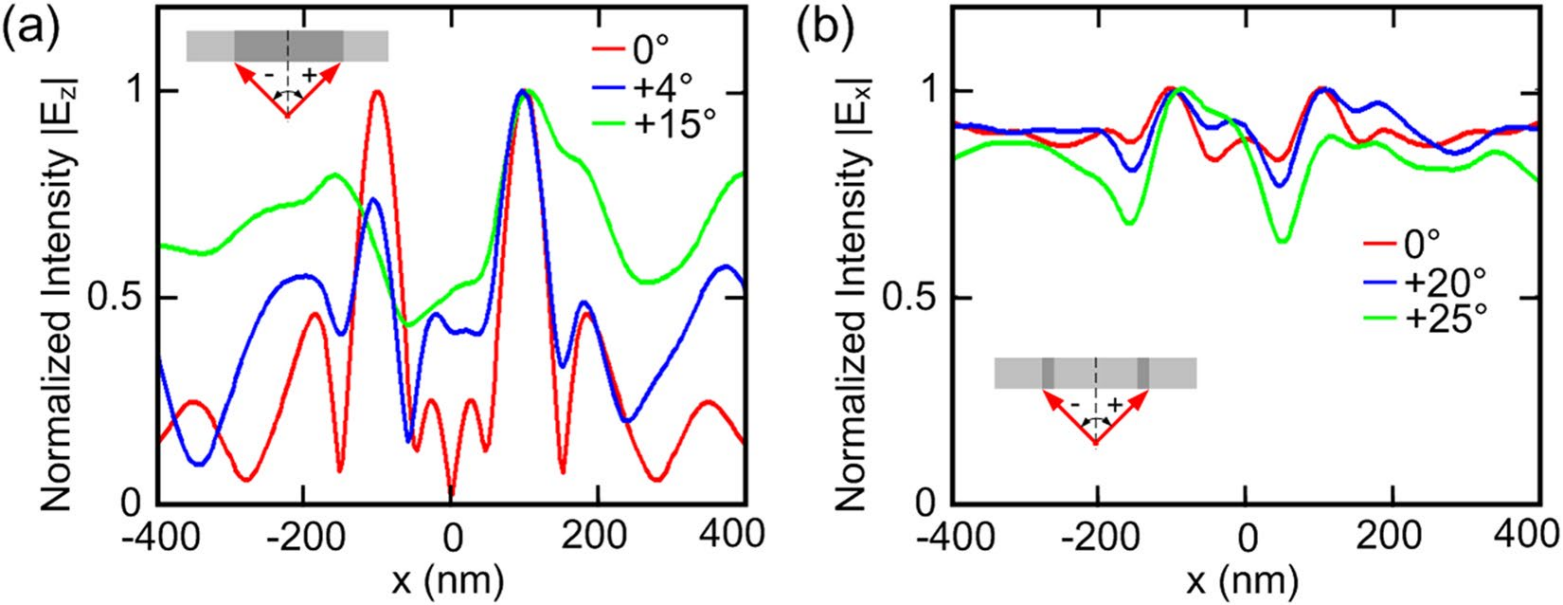
Characterization of the HPEDL for oblique incident light. (**a**) The HPEDL is utilized to detect the PMMA strip with 200 nm width that embedded in the PC substrate. (**b**) The HPEDL is utilized to detect two PMMA strips with 20 nm width and a center-to-center separation of 200 nm.

The above discussions are focused on normal incidence. To complete the analysis, we also characterize the ability of the detecting performance for oblique incidence. To facilitate comparison, we fix the HPEDL, the PMMA strips and the observation plane at their physical positions and use oblique incident light with angle varying from 0° to ±30°. For the sake of simplicity and due to the symmetry, only the line profiles of |*E*_*z*_| with varying angles along “+” direction are shown in Fig. 5a, “-” and “+” in the inset indicate the directions of oblique incident light. The HPEDL here is utilized to detect the PMMA strip with 200 nm width that embedded in the PC substrate. One can see that when the incident angle varies between 0° and +4° two-well separated peaks are still resolved, indicating that the detecting performance is still desirable. When the angle increases, from +5° to +15°, we could find only one peak that is located in the position of one interface. Further increasing angle, from +16° to +30°, this HPEDL will lose its efficacy. Similarly, we also show the results of the HPEDL when detecting two PMMA strips with 20 nm width and a center-to-center separation of 200 nm as shown in Fig. 5b. In this case, one can see that when the incident light angle varies from 0° to +20°, two separated peaks exist, indicating that the HPEDL can still detect the PMMA strips. When the incident angle increases to +25°, one peak disappears and the remaining peak is located in the position of one PMMA strip. When further increasing the incident angle to +30°, one can see that the HPEDL offers no information on sensitive detection.

**Figure 6:**
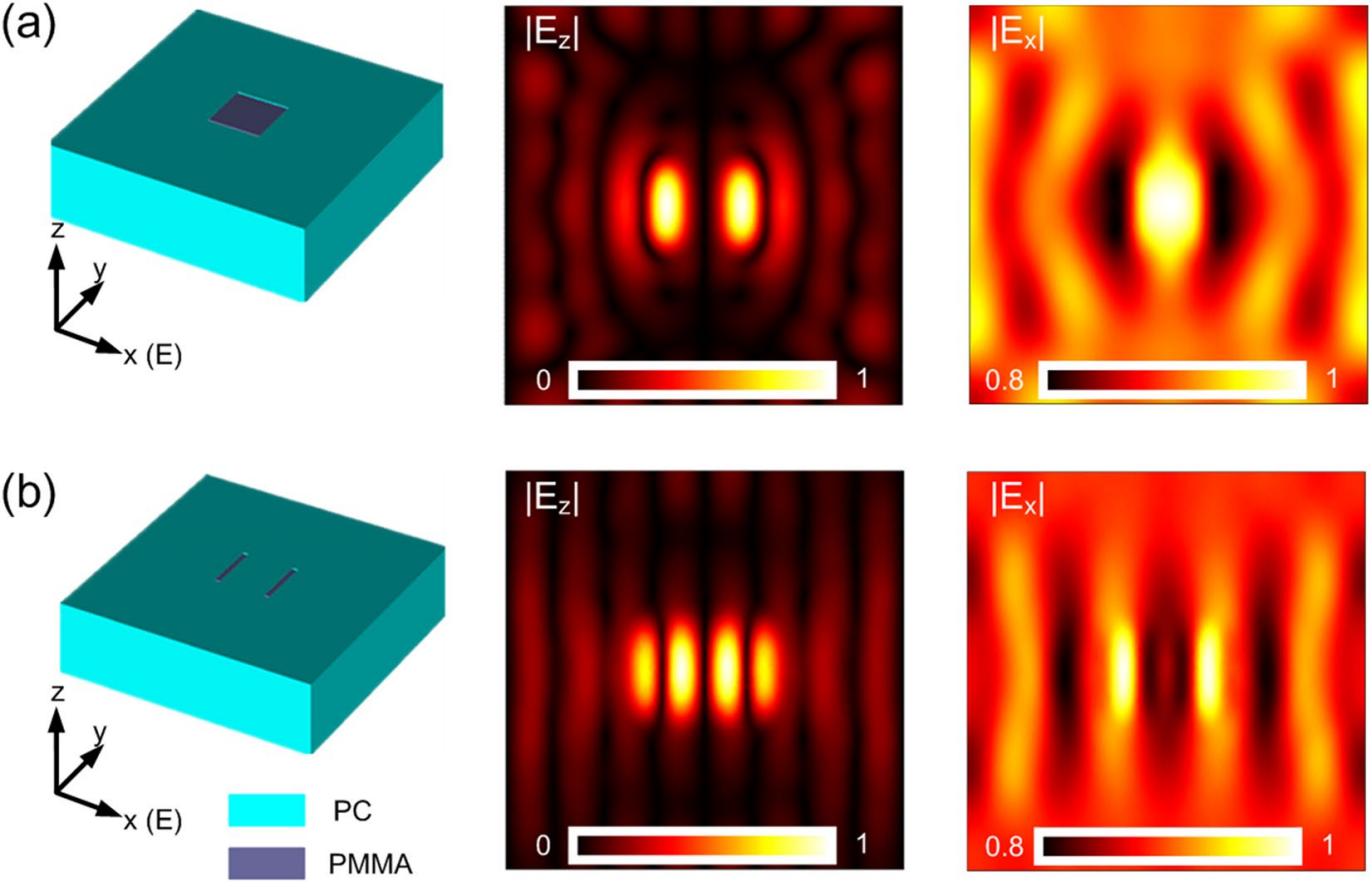
Detecting performance of the HPEDL in three-dimensional simulations. The HPEDL is used to detect two related patterns: (**a**) a square PMMA with 200 nm width; (**b**) two PMMA strips with 20 nm width and 200 nm long, the center-to-center separation of the strips is chosen to be 200 nm. The simulated electric-field distributions |*E*_*z*_| and |*E*_*x*_| for both structures are illustrated.

To further support the *straightforward* sensitive detection of the HPEDL, we extend the two-dimensional simulations to more intuitive and realistic three-dimensional cases. The HPEDL with thickness 320 nm is used to detect two patterns as sketched in the first column of Fig. 6: (i) a square PMMA with 200 nm width; (ii) two PMMA strips with 20 nm width and 200 nm long, the separation of the centers of the two strips is chosen to be 200 nm. In these simulations, normal incident light with the electric field oriented in the *x* direction is considered. The simulated electric-field distributions |*E*_*z*_| and |*E*_*x*_| at λ_0_ = 680 nm for both patterns are illustrated in the rest of Fig. 6, in which the second column is for |*E*_*z*_| and the third column is for |*E*_*x*_|. It can be seen that the interfaces of the square PMMA can be clearly resolved by measuring |*E*_*z*_| while the locations of two PMMA strips can be detected by probing |*E*_*x*_|, also in agreement with the two-dimensional simulations.

## Discussion

The previous research has shown that high-k modes induced inside the HMM will be cut off when the wave vector is close to the inverse size of the unit cell. This might in turn limit the degree of optical confinement and spatial resolution of HPs, and the HMM can no longer be treated as an effective medium at this situation. To completely exploit the potentialities of the HPs and improve the performance of sensitive detection, it is desirable to have the layers as thin as possible. However, growing noble metal films thinner than approximately 5−10 nm is challenging based on the state of the art of nanotechnology. Fortunately, photonic crystal slabs^35^ or naturally occurring materials^36, 37^, such as crystals with perovskite layer crystal structure (cuprate and ruthenate) and graphite or graphite-like materials (e.g., MgB_2_) have similar structures to that of the layered HMMs, regardless of different size scales of the layer. These media have attracted much attention recently in the realm of optical imaging and sensing and can be further utilized for sensitive detection.

Combining with the recent development of two-dimensional plasmons and metasurfaces^38–45^, the mechanism of sensitive detection could further extend to two-dimensional configuration. As indicated in ref. 45, hyperbolic dispersion topologies arise when the surface behaves as a dielectric (with Im[σ]<0) along one direction, and as a metal (with Im[σ]>0) along the orthogonal direction. An array of densely packed graphene strips can implement an ideal platform for the metasurface topologies at terahertz and near infrared frequencies. If we rotate the graphene strips by 90°, another type of hyperbolic metasurfaces can be achieved. By combining these two kinds of metasurfaces, we can realize a two-dimensional lens for detection.

To summarize, we have proposed a new lens design, which can manipulate hyperbolic polaritonic rays for *straightforward* sensitive detection. The lens could successfully achieve *straightforward* sensitive detection in a broadband spectral range with hyperbolic dispersion by modifying the thickness of each slab at different wavelengths. Compare with the traditional lens, the thickness modification makes the proposed lens design of greater adaptability and practicality. Furthermore, we investigate the minimum scale and the angular-dependent performance of the structure, which limits its applications to a certain degree. In spite of these, we still expect that this structure can serve as parts of a number of optical applications.

## Methods

The numerical calculations were performed using a Finite Element Method (FEM) solver, as implemented in the commercially available software package COMSOL.

## Electronic supplementary material

Supplementary Information
