# Supplementary material for: Hyperbolic-polaritons-enabled dark-field lens for sensitive detection
Source: Sci Rep. 2017 Aug 1;7:6995. doi: 10.1038/s41598-017-07576-z (PMC5539334; doi:10.1038/s41598-017-07576-z)
Supplement: Supplementary file 1 — Supplementary Information [file 41598_2017_7576_MOESM1_ESM.pdf]

SUPPLEMENTARY INFORMATION

Hyperbolic-polaritons-enabled dark-field lens for sensitive detection

Lian Shen[1,2], Huaping Wang[3], Rujiang Li[1,2], Zhiwei Xu[3], and Hongsheng Chen[1,2]

[1]State Key Laboratory of Modern Optical Instrumentations, Zhejiang University Hangzhou, 310027 China

[2]College of Information Science & Electronic Engineering, Zhejiang University, Hangzhou, 310027, China

[3]Institute of Marine Electronics Engineering, Zhejiang University, Hangzhou, 310058, China

In this Supplementary Information section, we provide the theoretical model of type I and type II hyperbolic metamaterials and the corresponding homogenized permittivities are computed. Furthermore we provide specific details on analytical solution of the plane wave transmission through the lens including the analytical evaluation of the wave impedances at the boundaries and the derivation of the elements of the permittivity tensors for the phase compensation condition.

S1. Theoretical model of type I and type II hyperbolic metamaterials (HMMs)

Here we provide additional details for the theoretical model of type I and type II HMMs. A type I HMM has a negative permittivity in the *z*-direction (along light propagation) and positive permittivity in the *x*-*y* directions can be realized, for example, with plasmonic nanowire arrays. Plasmonic nanowire arrays represent collections of aligned metallic wires (For example, gold with permittivity ε_{Au} and average radius *r*) embedded into a dielectric host (For example, Al_2O_3 with permittivity $\varepsilon_{Al_2O_3}$) with mean center-to-center separation a. Nanowire metamaterials typically operate in the effective medium regime where $r < a \ll \lambda_0$ with λ_0 being the free-space wavelength. In this regime, the optical properties of the composite can be well described by averaged geometric parameters (concentration $p = \pi r^2/a^2$) and are weakly affected by the details of wire distribution. In the quasistatic limit, the Maxwell-Garnett effective medium theory[1,2] predicts the following relation between the effective permittivity, the permittivities of the

components of the composite, and the geometrical parameters of the metamaterial,

$$\varepsilon_{x,y} = \varepsilon_{Al_2O_3} \frac{(1+p)\varepsilon_{Au} + (1-p)\varepsilon_{Al_2O_3}}{(1+p)\varepsilon_{Al_2O_3} + (1-p)\varepsilon_{Au}}, \varepsilon_z = p\varepsilon_{Au} + (1-p)\varepsilon_{Al_2O_3}. \quad \text{(S1)}$$

For simplicity, we keep the filling fraction $p = 0.3$. We consider Au as metallic rod and Al_2O_3 as the dielectric host and for their permittivities, we use

$$\varepsilon_{Au} = \varepsilon_1 - \frac{\omega_p^2}{\omega^2 + i\Gamma_p\omega} + \sum_{m=1}^{n} \frac{f_m\omega_m^2}{\omega_m^2 - \omega^2 - i\Gamma_m\omega}, \quad \text{(S2)}$$

with $\mathrm{n} = 1$, $\varepsilon_1 = 6.8890, \omega_p = 8.9601\mathrm{eV}$, $\Gamma_p = 0.0723\mathrm{eV}$, $\omega_1 = 2.9715\mathrm{eV}$, $f_1 = 1.7857$ and $\Gamma_1 = 0.9503\mathrm{eV}$. The parameters of Drude-Lorentz model with 1 Lorentz oscillator for gold are obtained by fitting to the data from Ref. [3]. The dielectric host is assumed to be a permittivity of $\varepsilon_{Al_2O_3} = 3$[3]. The real and imaginary parts of the effective medium parameters of the plasmonic nanowire arrays are illustrated in Figure S1.

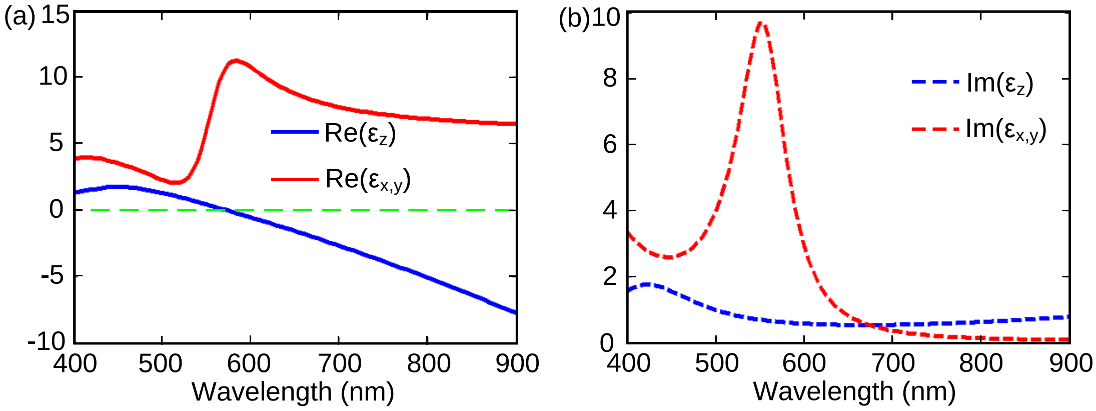

Figure S1| Dielectric functions of a type I hyperbolic metamaterial. (a) Real and (b) imaginary parts of the dielectric functions of gold nanorod arrays embedded into the Al_2O_3 host. The gold nanorod arrays can be considered as type I hyperbolic media in the spectral range 575-900 nm. Green dashed lines separate the positive and negative values of the real parts of the dielectric functions.

Likewise, a type II HMM can be realized by alternating layers of metal/dielectric thin films, producing a negative permittivity in the *x*-*y* directions and a positive permittivity in the *z*-direction. By considering the optical properties of the constituent materials and their filling fractions f_m, $f_m = a_m/(a_m + a_d)$, where a_m and a_d are the thickness of individual layer of metal and dielectric, respectively) the effective material permittivities of the entire structure are obtained by effective medium theory[4,5],

$$\varepsilon_{x,y} = f_m\varepsilon_{Ag} + (1 - f_m)\varepsilon_{SiO_2}, \varepsilon_z = \frac{\varepsilon_{Ag}\varepsilon_{SiO_2}}{(1-f_m)\varepsilon_{Ag}+f_m\varepsilon_{SiO_2}}. \quad \text{(S3)}$$

The filling fractions f_m in the main text is chosen to be $f_m = 0.5$ ($a_m = a_d = 10$nm). We consider silver as the metal layers, and for its permittivity, we use Drude-Lorentz model with 1 Lorentz oscillator

$$\varepsilon_{Ag} = \varepsilon_1 - \frac{\omega_p^2}{\omega^2+i\Gamma_p\omega} + \sum_{m=1}^{n} \frac{f_m\omega_m^2}{\omega_m^2-\omega^2-i\Gamma_m\omega}, \quad \text{(S4)}$$

with $\mathrm{n} = 1$, $\varepsilon_1 = 3.7180, \omega_p = 9.2093\mathrm{eV}$, $\Gamma_p = 0.02\mathrm{eV}$, $\omega_1 = 4.2840\mathrm{eV}$, $f_1 = 0.4242$ and $\Gamma_1 = 0.3430\mathrm{eV}$. The parameters are obtained by fitting to the data from Ref. [3]. The dielectric layer is assumed to be a permittivity of $\varepsilon_{SiO_2} = 2.25$[3]. Figure S2 shows the corresponding effective medium parameters of a silver-silica lamellar structure with a metallic filling fraction of $f_m = 0.5$.

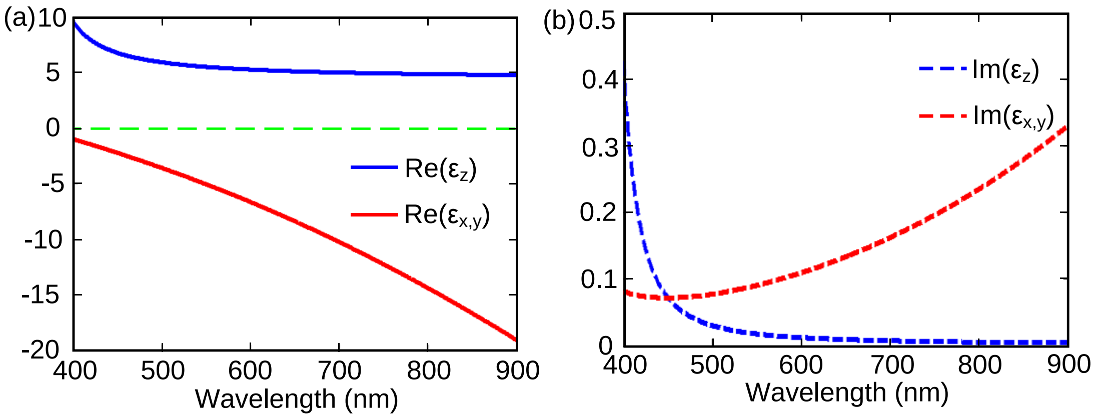

Figure S2| Dielectric functions of a type II hyperbolic metamaterial. (a) Real and (b) imaginary parts of the dielectric functions of a silver-silica lamellar structure. The thickness of silver and silica layers are 10 nm and 10 nm, respectively. The lamellar structure can be considered as type II hyperbolic media in the spectral range 400-900 nm. Green dashed lines separate the positive and negative values of the real parts of the dielectric functions.

S2. Analytical calculations of transmission coefficient of the HPEDL

From Figure S3, we find that the schematic configuration is illuminated by a transverse magnetic polarized incident field. The HPEDL consisting of type I ($\mathrm{Re}[\varepsilon_{x,y}] > 0, \mathrm{Re}[\varepsilon_z] < 0$) and type II ($\mathrm{Re}[\varepsilon_{x,y}] < 0, \mathrm{Re}[\varepsilon_z] > 0$) hyperbolic media can be utilized to detect the weakly scattering nanoscale features.

The plane wave transmission coefficient is

$$T = 2Z_0Z_1Z_2 \begin{Bmatrix} Z_0[2Z_1Z_2 \cos\phi_1 \cos\phi_2 - (Z_1^2 + Z_2^2)\sin\phi_1 \sin\phi_2] \\ -i[Z_1(Z_0^2 + Z_2^2)\cos\phi_1 \sin\phi_2 + Z_2(Z_0^2 + Z_1^2)\sin\phi_1 \cos\phi_2] \end{Bmatrix}^{-1}, \quad \text{(S5)}$$

where $Z_1 = k_{1z}/(\omega\varepsilon_{1x}\varepsilon_0)$ and $Z_2 = k_{2z}/(\omega\varepsilon_{2x}\varepsilon_0)$ represent the characteristic impedances of type I and type II hyperbolic media, $Z_0 = k_{0z}/(\omega\varepsilon_0)$ is the impedance of the surrounding medium (assumed to be free space, the surrounding medium with closed isofrequency surface will not influence the transmission coefficient of the high-k field waves), ε_0 is the permittivity of free space. The phase advances in two media are $\phi_1 = k_{1z}d_1$ and $\phi_2 = k_{2z}d_2$, respectively, and the temporal dependency is $\exp(-i\omega t)$.

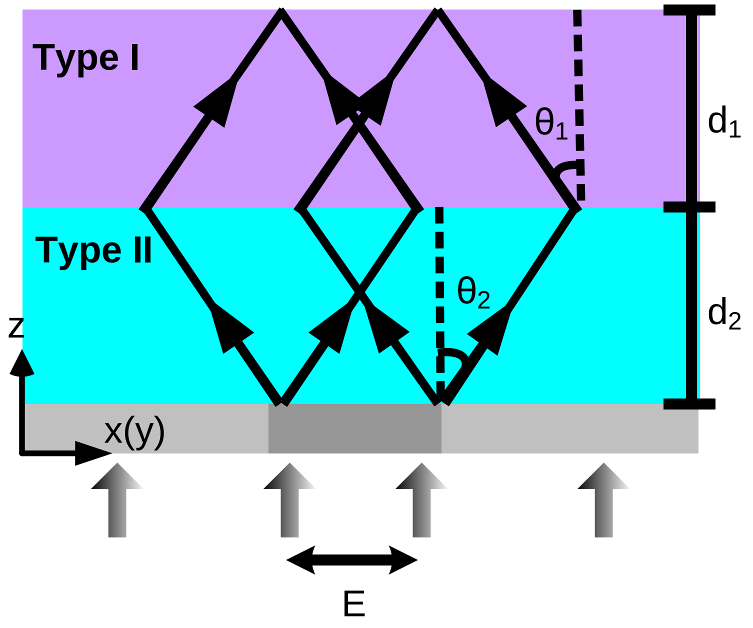

Figure S3| The operation principle of the HPEDL. θ_1 and θ_2 are the angles between the Poynting vector (dark arrows) and the optical axis for type I and type II hyperbolic media, respectively.

The dispersion relations in the two media have

$$\frac{k_{1z}^2}{\varepsilon_{1x}} + \frac{k_x^2}{\varepsilon_{1z}} = k_0^2 = \frac{\omega^2}{c^2}, \frac{k_{2z}^2}{\varepsilon_{2x}} + \frac{k_x^2}{\varepsilon_{2z}} = k_0^2 = \frac{\omega^2}{c^2}, \quad \text{(S6)}$$

At high k approximation, $k_x^2 \gg |\varepsilon_z|k_0^2$, $k_{1z} \approx k_x\sqrt{-\varepsilon_{1x}/\varepsilon_{1z}}$ and $k_{2z} \approx -k_x\sqrt{-\varepsilon_{2x}/\varepsilon_{2z}}$. The allowed directions for the momentum or the phase velocity of the high-k field waves in both media are respectively restricted to the angle $\varphi_{1c} = arctan(\sqrt{\varepsilon_{1z}}/i\sqrt{\varepsilon_{1x}})$ and $\varphi_{2c} = arctan(\sqrt{\varepsilon_{2z}}/i\sqrt{\varepsilon_{2x}})$ with respect to the optical axis. Since the Poynting vector and group velocity of HPs are orthogonal to the momentum, the propagation direction of the HPs has a fixed

angle $\theta = \pm(\pi/2 - \varphi_C)$ with respect to the z-axis.

For high-k field waves, the phase advances and the characteristic impedances in these two media become

$$\phi_1 \approx k_x d_1 \sqrt{-\varepsilon_{1x}/\varepsilon_{1z}} = k_x d_1 \tan\theta_1, \phi_2 \approx -k_x d_2 \sqrt{-\varepsilon_{2x}/\varepsilon_{2z}} = -k_x d_2 \tan\theta_2, \quad \text{(S7)}$$

$$Z_1 \approx \frac{k_x}{\omega\varepsilon_0\sqrt{-\varepsilon_{1x}\varepsilon_{1z}}}, Z_2 \approx \frac{k_x}{\omega\varepsilon_0\sqrt{-\varepsilon_{2x}\varepsilon_{2z}}}. \quad \text{(S8)}$$

When the parameters satisfying

$$d_1\sqrt{-\varepsilon_{1x}/\varepsilon_{1z}} = d_2\sqrt{-\varepsilon_{2x}/\varepsilon_{2z}}, \varepsilon_{1x}\varepsilon_{1z} = \varepsilon_{2x}\varepsilon_{2z}, \quad \text{(S9)}$$

we could deduce that $Z_1 = Z_2$ and $\phi_1 + \phi_2 = 0$, indicating that both impedance match condition and phase compensation condition are satisfied. In this situation, the transmission coefficient becomes $T \approx 1$. Generally, low-k field waves encode large geometric features, while high-k field waves describe finer details. Since the transmission coefficient is close to unity for high-k field waves, subwavelength features could be effectively transferred through the HPEDL.

As discussed in Part S1, we find that in the overlapping spectral range 575-900 nm, both type I and type II HMMs are realizable. Since both effective parameters are dispersive, in order to meet the impedance match condition, we need rigorous effective parameters, which make the realization of this HPEDL more difficult. We are willing to see that the impedance match condition will not affect the transmission coefficient so much when the phase compensation condition satisfies. Since the material loss is very large in the spectral range 575-600 nm, only a small part of waves can be transmitted through the structure. Figure S4 shows the transmission coefficient in the spectral range 600-900 nm, the thickness of each media is modified in order to meet the phase compensation condition. From the Figure S4, we could find that the transmission coefficient of the high-k field waves is close to unity in the spectral range 600-900 nm. This phenomenon indicates that the phase compensation condition plays an important role in sensitive detection.

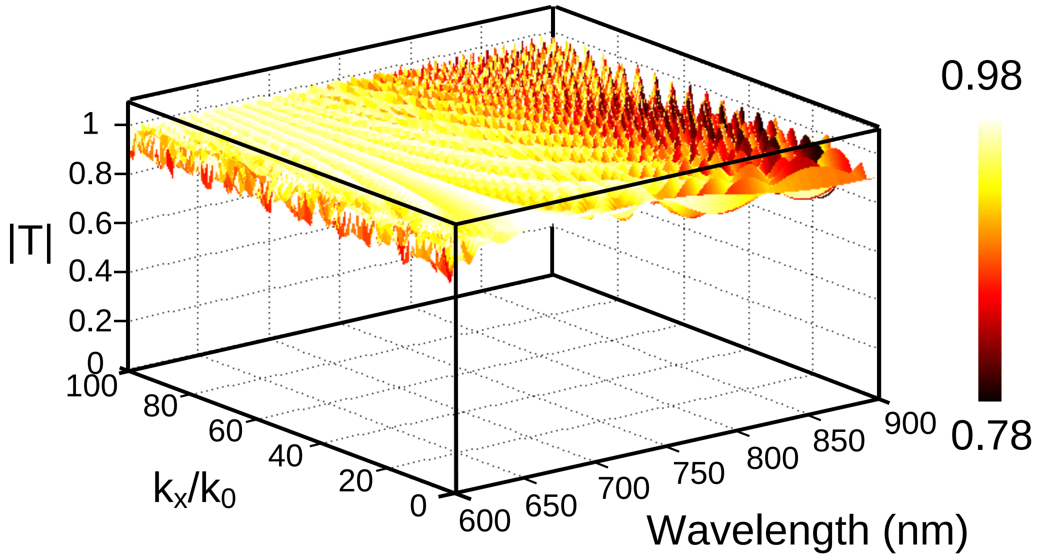

Figure S4| The transmission coefficient in the spectral range 600-900 nm. The thickness of each media is modified such that the phase compensation condition is satisfied.

References

1. Atkinson, R. *et al.* Anisotropic optical properties of arrays of gold nanorods embedded in alumina. *Phys. Rev. B: Condens. Matter*, **73**, 235402 (2006).
2. Lagarkov, A. N. & Sarychev, A. K. Phys. Rev. B, **53**, 6318 (1996).
3. Johnson, P. B. & Christy, R. W. Electromagnetic properties of composites containing elongated conducting inclusions. *Phys. Rev. B: Solid State*, **6**, 4370–4379 (1972).
4. Landau, L. D., Lifshitz, E. M. & Pitaevskii, L. P. *Course of Theoretical Physics*, 2nd ed. Reed, Oxford (1984).
5. Brekhovskikh, L. M. *Waves in Layered Media*, 2nd ed. Academic, New York (1980).
